# Health Outcomes in EU Cross-Border Regions: A Scoping Review

**DOI:** 10.3389/phrs.2025.1608170

**Published:** 2025-02-24

**Authors:** Sophie Stroisch, Viola Angelini, Sebastian Schnettler, Tobias Vogt

**Affiliations:** ^1^ Faculty I - Educational and Social Sciences, Institute for Social Sciences, Carl von Ossietzky University of Oldenburg, Oldenburg, Germany; ^2^ Population Research Centre, Faculty of Spatial Sciences, University of Groningen, Groningen, Netherlands; ^3^ Department of Economics, Econometrics and Finance, Faculty of Economics and Business, University of Groningen, Groningen, Netherlands; ^4^ Healthy Demography Centre, Prasanna School of Public Health, Manipal Academy for Higher Education, Manipal, India

**Keywords:** scoping review, European Union, public health, morbidity, mortality, cross-border

## Abstract

**Objective:**

This scoping review examines health outcome trends in European cross-border regions, identifies available evidence, and highlights research gaps. The European Union’s integration efforts aim to harmonise living standards and healthcare access. Removed border controls and freedom of movement enhanced service availability, benefiting populations in border regions with cross-border healthcare access. However, these populations are exposed to different institutional settings, highlighting health differences worth studying.

**Methods:**

We employed the Joanna Briggs Institute methodology, using the PCC (Population-Concept-Context) framework to set eligibility criteria. The search covered literature databases and international governmental institution websites, yielding 785 studies, with 24 included in the final analysis.

**Results:**

No comprehensive studies investigating longitudinal population health patterns were found. Instead, there are studies on specific diseases or health outcomes in particular border regions, especially around Germany. Most of these studies were cross-sectional. Five key research themes emerged: antibiotic resistance, COVID-19/SARS-CoV-2, other infectious diseases, cancer survival, and additional health outcomes.

**Conclusion:**

The findings suggest that cross-border contexts have predominantly been used to study infectious disease spread, with little attention given to the broader impact of European integration on long-term health trends.

## Introduction

### Rationale

Europe has witnessed consistent improvements in living standards, working conditions, and cross-border collaborations [1, 2] over the past 30 years, in part thanks to the ongoing process of European integration aiming to promote unity and solidarity across the continent [3]. The Maastricht Treaty, signed in 1992, played a significant role in this integration by establishing the European Union (EU) and promoting the elimination of border controls. Member states have experienced economic growth since joining the EU [4, 5]. This economic growth, in turn, positively impacted population health, as an increase in GDP *per capita* is associated with an increase in life expectancy in the long run [6–9]. The removal of hard borders strengthened economic, political, and cultural exchange, contributing to the harmonisation of living conditions. The freedom to live and work in other member states and to use their infrastructure, such as healthcare, may have impacted overall life satisfaction, which in turn has a positive effect on health [10, 11].

Besides these potential indirect effects of European integration processes on health outcomes, reducing health inequalities within and between regions is also an explicit and central aim of the EU. With Directive 2011/24/EU [12], citizens can receive healthcare across member states without further costs, to converge health differences and synchronize healthcare among EU member states. Meanwhile, the EU provides structural funds, such as the European Regional Development Fund, to help disadvantaged regions address imbalances between them. Notably, the Interreg project is instrumental in fostering regional development, strengthening cohesion, and reducing economic disparities through cross-border collaboration [13]. Increased cooperation and the resulting knowledge exchange make border regions key contributors to European integration. Cross-border partnerships in the health sector exemplify the EU’s efforts toward harmonisation, as reflected in Directive 2011. However, despite these efforts, synchronising social and healthcare in the EU remains a challenge due to the fundamental differences between the systems in each state [14]. These differences are unlikely to change since the systems are deeply rooted in the fundamental structure of the states.

The COVID-19 pandemic has highlighted the critical need for improved cross-border health policies, revealing gaps in coordination and resource sharing [15]. In response, the EU introduced the European Health Union initiative [16], which aims to strengthen health coordination during crises and improve the resilience of healthcare systems across member states. These measures are designed to better prepare the EU for future cross-border health threats and pandemics while addressing disparities in healthcare provision. Cross-border regions offer a unique setting to explore health differences within the framework of European integration. These regions function as natural laboratories for studying the impact of varying policies and healthcare systems on health outcomes. Despite sharing cultural and historical similarities, populations on either side of a border operate within distinct institutional environments, including healthcare systems. Moreover, residents often benefit from access to infrastructure and services in neighboring countries.

Many European border regions are situated in rather remote and rural parts of the country, often far away from national central hubs of economic activity. This peripheral positioning is often characterised by limited development in terms of infrastructure, transportation, and overall accessibility. Consequently, these regions may have faced economic stagnation and a steady population decline, as residents migrated to urban centres in search of better opportunities [17]. Declining birth rates and high levels of out-migration have resulted in shrinking and ageing populations, leading to an uneven distribution of age groups and straining the local labour markets [18]. The impact of these dynamics is particularly evident in Eastern and Central Europe, where non-metropolitan regions have been experiencing pronounced population declines [19].

The core-periphery concept suggests that peripheral regions become dependent on central regions [20]; however, this can be reversible due to economic growth and the reorganization of activities in space [21–23]. Due to European integration and globalisation, cross-border areas might have shifted from those dependent peripheries to regions that now draw in new industries [24]. In fact, the opening of national borders has contributed to an increase in regional economic activity for border regions within Europe [25], particularly metropolitan border areas in Western Europe benefitted from better connectivity and economic prospect.

Insights on how these changes have impacted the health of cross-border populations over time remain limited in the literature. Existing research on health in Europe has conventionally emphasized within- or between-country comparisons, whereas these comparisons have rarely gone beyond national borders. While there is an ever-growing literature on health and morbidity at the sub-national level within the European Union [26–28], most of these studies are confined to provincial (NUTS-2) levels and do not specifically address (cross-) border regions. This limitation has hindered a comprehensive understanding of health outcomes on a regional scale across different countries. Therefore, it is imperative to broaden the scope of research to include cross-border comparisons that can provide insights into the drivers of the differences and similarities in health outcomes between regions. Such an approach would enable policymakers to develop more tailored and effective health policies that are better aligned with regional needs.

### Objectives

Our scoping review seeks to systematically map the presence of empirical studies focusing on the differences and trends of health outcomes over time within various health policy frameworks for individuals residing in cross-border regions among EU and Schengen area member states. A comprehensive overview of health indicators in European cross-border areas since the establishment of the EU has not yet been conducted. We anticipate that such an overview could guide future research on health in EU cross-border regions by identifying evidence sources, cross-border data availability, and literature gaps. In our review, we focused on the following guiding questions:

a) Since the Maastricht Treaty was implemented in 1992, what is known regarding the differences and developments in health outcomes within EU cross-border regions?
b) What empirical evidence and data are available regarding differences in health across borders?
c) What gaps can be observed in the existing literature?

## Methods

Due to the multidisciplinary and the diversity of the literature on health in cross-border regions, a scoping review is a suitable approach for this paper. We conducted an initial search of PubMed/MEDLINE, the Cochrane Database of Systematic Reviews, and Joanna Briggs Institute Evidence Synthesis. However, we did not discover any existing or ongoing systematic or scoping reviews related to the subject.

We followed the Joanna Briggs Institute methodology for scoping reviews [29, 30]. Additionally, we applied the Preferred Reporting Items for Systematic Reviews and Meta-Analysis extension for Scoping Reviews (PRISMA-ScR) as a framework to assist us in addressing the research question at hand [31]. The PRISMA-ScR checklist is provided in the Supplemental Material. In the following, we provide a summary of our inclusion criteria and methods for this scoping review. For more details, see our research protocol [32]. Following the suggestion of Pieper and colleagues, we reused text from our protocol in the introduction and method sections, as the research objectives and the methodology remained largely unchanged from the original plan [33].

### Selection Criteria

We considered peer-reviewed articles as well as book chapters, policy reports, working papers, or organizational reports for inclusion in this review. Our search was limited to studies published exclusively in English. Furthermore, we disregarded studies published before 1992, that is, studies predating the Maastricht Treaty enactment in 1992 and its subsequent implementation in 1993. The selection criteria for inclusion and exclusion were developed under the Population, Concept, and Context (PCC) approach recommended by PRISMA-ScR.

First, the population of this scoping review encompassed all residents of varying age groups residing in cross-border regions of all EU member states and Schengen area countries. Second, the central concept was to explore disparities or similarities in health outcomes among the cross-border population in the European Union and how these outcomes have evolved. Health outcomes comprised mortality (e.g., life expectancy, survival rates), morbidity (e.g., disease prevalence, incidence rates), and measures of disease burden, which combine aspects of both morbidity and mortality. Third, our review focused on the context of European integration.

The Nomenclature of Territorial Units for Statistics (NUTS) classification, established by Eurostat and the European Commission, defines border regions as NUTS-3 areas that either share a land border or have more than half of their population living within 25 km of the border. However, limiting our search to a small scale would have excluded relevant studies that were conducted on a larger geographical scale. Therefore, we have also included studies that consider their study location as cross-border, even if it is at a larger regional scale, such as NUTS-2 level. The key requirement was that it must be at a subnational level so that a border region can be differentiated from the rest of the country.

### Search Strategy and Data Management

We conducted a three-step search strategy in alignment with the PRISMA-ScR. After a preliminary search on PubMed and Scopus, we refined our search with updated keywords and index terms on the databases of PubMed, Scopus, Web of Science and SocIndex (EBSCOhost). We conducted a preliminary search in August 2022, followed by two updates in August 2023 and September 2024, to account for papers that have been published after our initial search. The search syntax was developed using three key terms based on the PCC framework: “cross-border” (Population), “European Union” (Context), and “health outcome” (Concept). For each key term, we included several related keywords and, when appropriate, relevant index terms. We also adapted the syntax for each database according to its specific syntax requirements. The complete search strategy syntax of all databases is provided in the Supplementary Material (See Supplementary Material S1 – Syntax and Key Terms). Furthermore, we examined reference lists of included articles and searched the publication websites of the World Health Organisation, the EU, and the Organization for Economic Co-operation and Development (OECD) for additional studies. Duplicates were identified and systematically removed with the help of the citation manager Zotero (V.6.0.29, 2023). Two independent reviewers assessed the titles and abstracts using the Rayyan software to ensure they met the eligibility criteria for this review. The full text of the selected articles was thoroughly assessed. Disagreements between the reviewers were resolved through joint discussion.

### Data Analysis

After the selection process had been completed, we extracted the data from the final included studies. As proposed in our protocol, we extracted the following information: “Author,” “Location,” “Period (Year),” “Population,” “Method,” “Variable,” “Results,” “Data source,” and “Research Gaps.” We added the “Research Objective” to the table. The result chapter includes an abbreviated version of the table. Furthermore, we summarized evidence from the studies in the form of summary statistics, data visualisations (using R and Tableau Desktop), and narrative text.

## Results

### Study Selection

**FIGURE 1:**
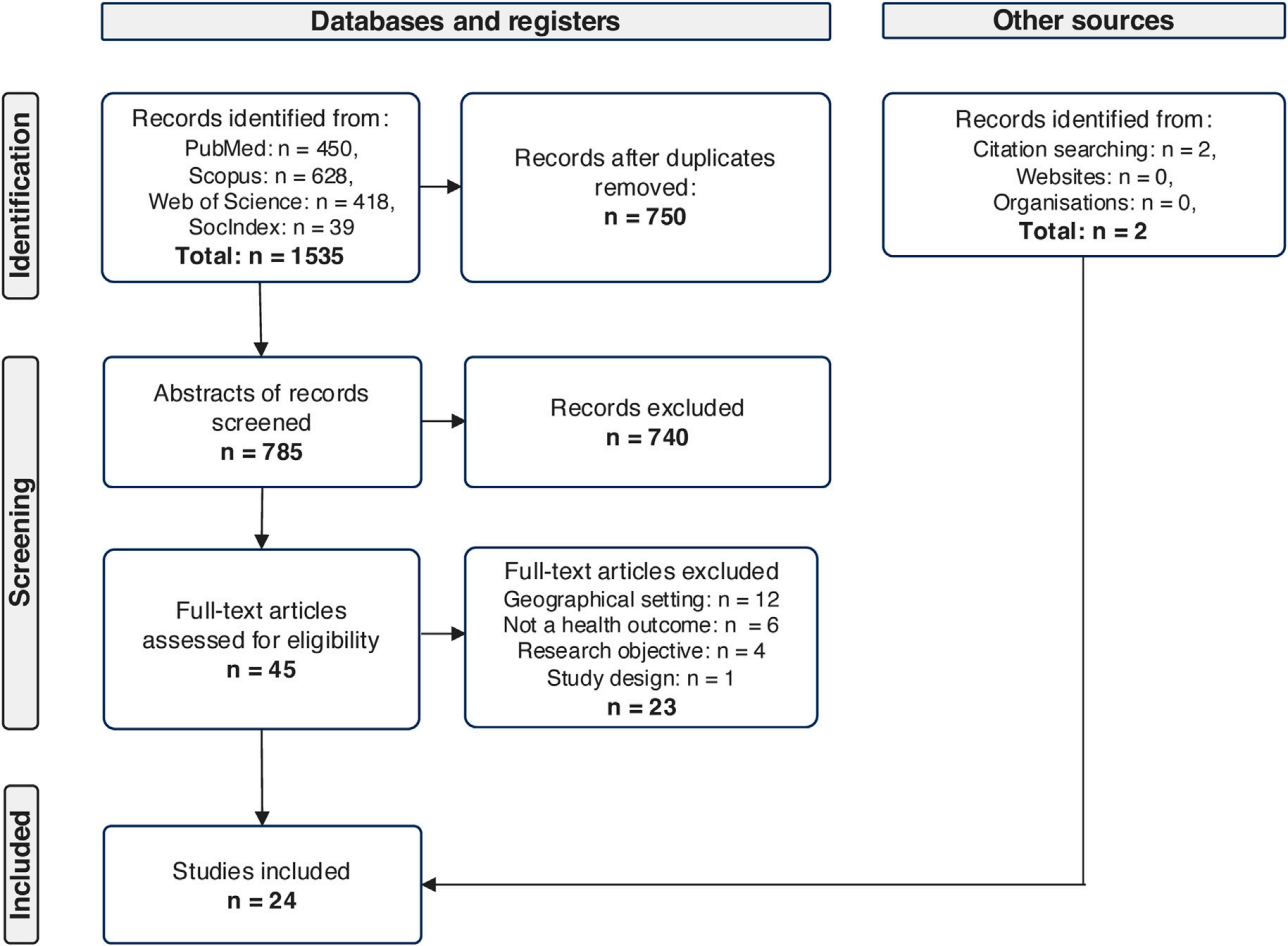
Flow diagram according to the Preferred Reporting Items for Systematic Reviews and Meta-Analysis extension for Scoping Reviews; based on Moher et al., 2009 (Netherlands, 2024).

A comprehensive search was conducted across four databases, identifying a total of 1,535 records. After removing 750 duplicate records, the remaining 785 were screened based on their titles and abstracts. A total of 740 records were excluded based on our screening criteria. We then carefully analysed the full text of 45 articles and removed any records that did not meet our eligibility requirements. Reasons for exclusion included lack of regional focus, studies limited to one side of the border, and non-health-related outcome variables. To ensure a thorough search, we also examined websites, publication databases of European and health organizations, and reference lists of retrieved articles, which led to the inclusion of two additional records. The scoping review ultimately included 24 studies. Figure 1 displays the PRISMA-flow diagram, illustrating the sequential selection and exclusion of studies.

### Characteristics of the Articles

Most of the selected studies (n = 15) were cross-sectional epidemiological studies focusing on infectious diseases in a specific cross-border setting of two or three countries. Meanwhile, we did not detect comprehensive studies investigating health patterns over time on a macro level for several cross-border regions. Although we found studies that investigated differences in other health parameters such as cancer survival or physical health indicators, these were also limited temporally and geographically. We classified the results into five thematic groups based on the health parameters examined by the studies: Antibiotic resistance, COVID-19/SARS-CoV-2, other infectious diseases, cancer survival, and other health outcomes.

#### Year of Publication

The selected articles for review were published between 2005 and 2024. We did not find any article between the implementation of the Maastricht Treaty in 1992 and 2004. Only a few studies emerged in the 2000s and the number of studies published per year fluctuated. There were minor rises in 2015 and 2022, during which four and three studies were published, respectively. At the same time, we have seen an increase in studies since 2020, mainly due to the emergence of COVID-19 studies.

#### Temporal Coverage of the Data

**FIGURE 2:**
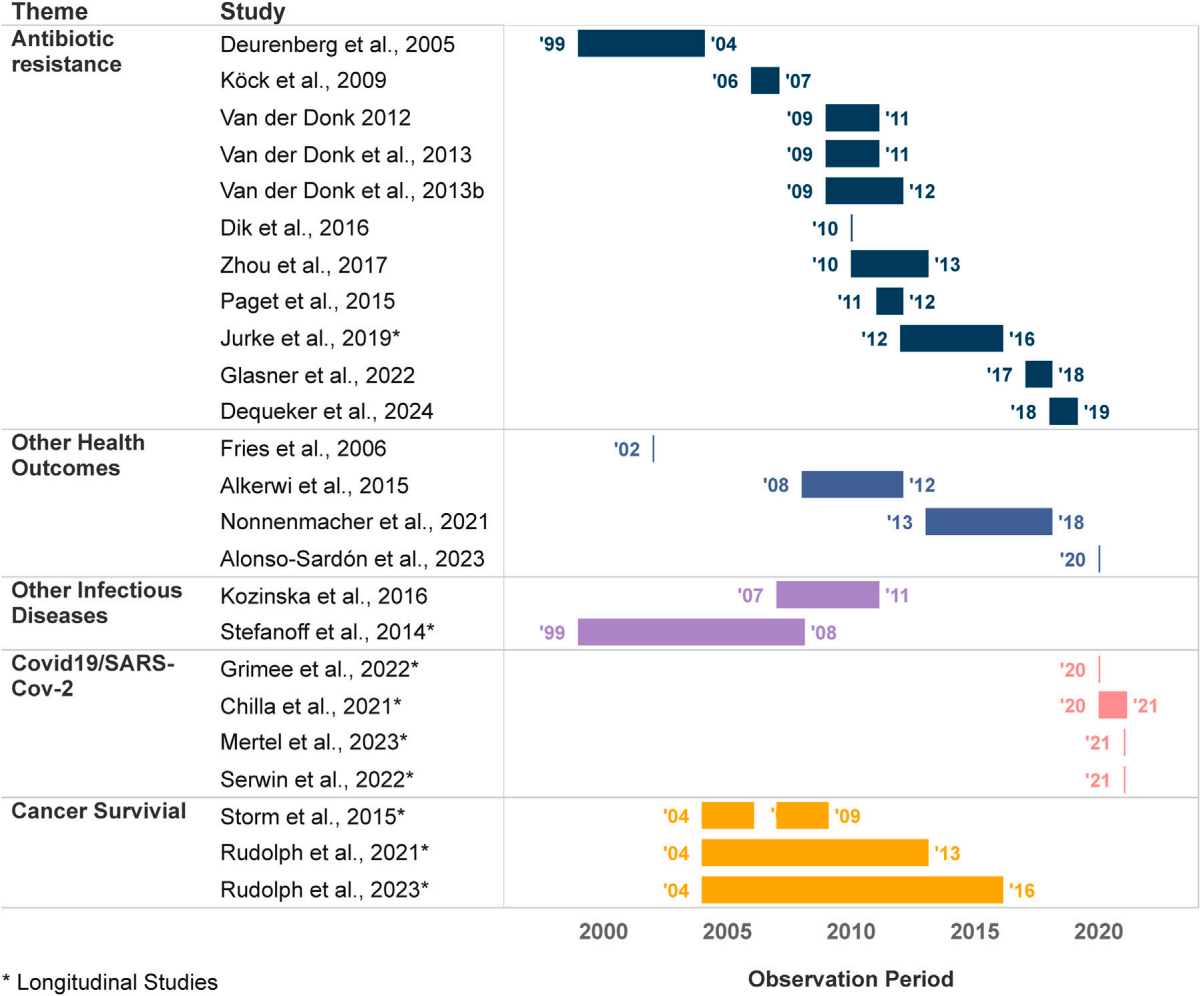
Temporal Coverage: Observation or data collection period of the selected articles (Netherlands, 2024).

Many of the eligible articles were cross-sectional epidemiological studies that focus on infectious diseases in a specific cross-border setting of two or three countries (see Figure 2 below for an overview of the respective observation windows). The earliest year of data utilization was in 1999 and the last in 2018 for studies on antibiotic resistance. Investigations on transnational cancer research were carried out during the interval spanning from 2004 to 2016. Subsequently, data commencing from the year 2020 were used in the context of studies about COVID-19 and SARS-CoV-2. Nine out of 24 used a longitudinal design with a diverse observation window. While COVID-19 studies use an observation period of up to 1 year, studies on cancer survival and other infectious diseases observe data for up to 9 years.

#### Spatial Coverage of the Data

**FIGURE 3:**
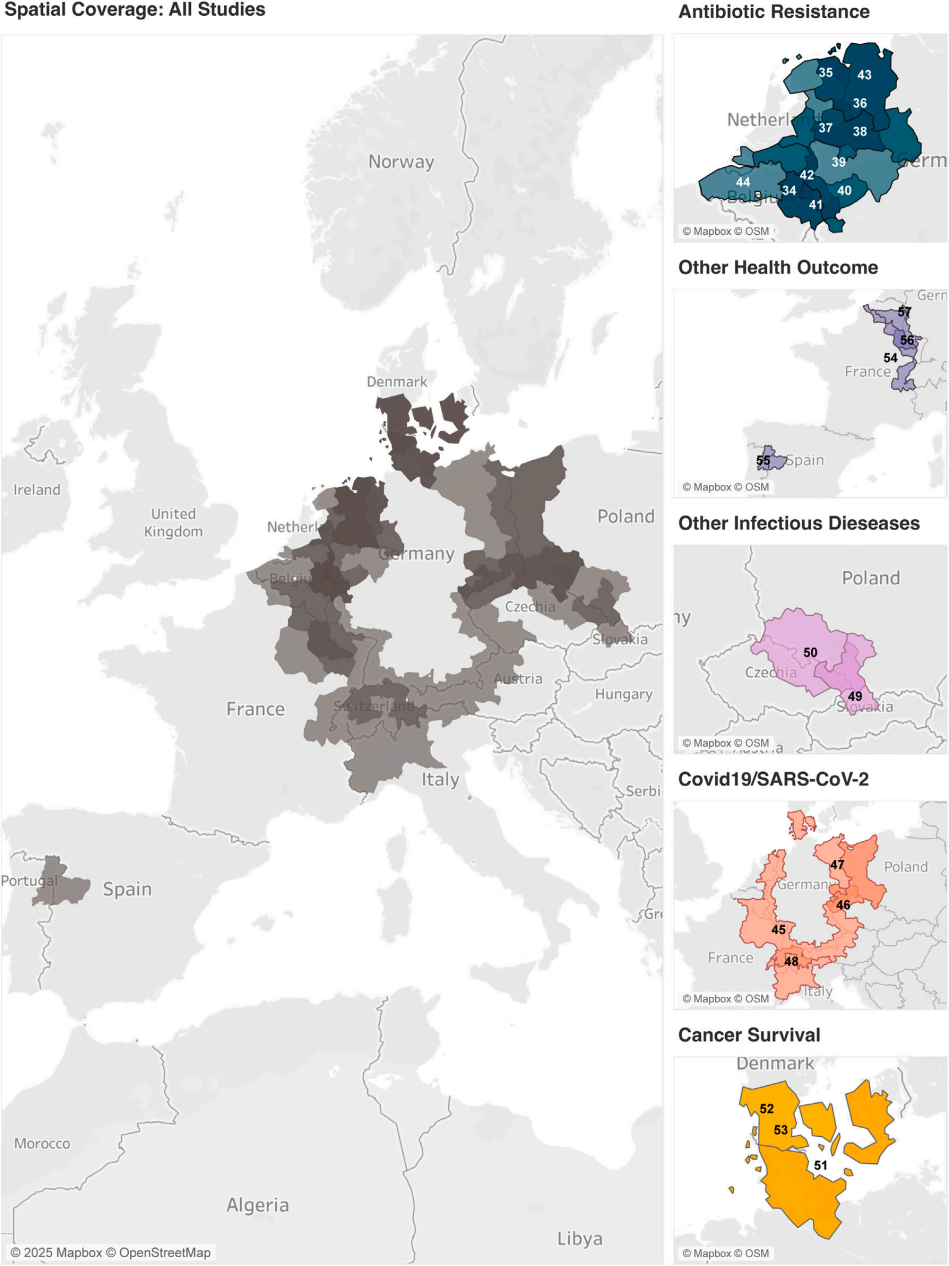
Geographical coverage of the selected studies. The map on the left summarises all studies in the scoping review. The maps on the right side are categorised according to the respective themes. The darker the shade, the more studies were conducted in the respective region. The numbers refer to the ID as indicated in Table 1 (Netherlands, 2024).

Figure 3 shows that the studies selected for analysis primarily focus on Western Europe. Of these, many studies (17 out of 24) cover research on Germany and its neighbouring countries only, with a particular clusteringin West-Germany, alongside the Dutch and Belgian border regions, of which the majority are studies on antibiotic resistance. Another regional cluster of research can be observed in the Fehmarn-Belt region, the border area between Denmark and Germany, where three cross-border studies on cancer survival were conducted. Studies in cross-border regions of Poland, Czechia, and Slovakia deal with the spread of other infectious diseases such as *Mycobacterium tuberculosis* and Tick-Borne Encephalitis and Lyme Borreliosis. One study on COVID-19 covered all German border regions, including those in neighbouring countries. Two additional COVID-19 studies were conducted in the Polish-German and Czech-German border regions, respectively, while one more study was conducted in Switzerland and Northern Italy. Other health outcomes have also been examined, such as the prevalence of neurodegenerative diseases in a Spanish-Portuguese cross-border region and the outcomes of cardiopulmonary resuscitation along the Dutch-Belgian-German border region.

#### Data Sources

Microbiological data in many studies were collected through swabs taken from patients in participating healthcare institutions. This type of data collection was dominant in studies on antibiotic resistance. In some cases, questionnaires or patient documentation were used to supplement the data. Primary data was also collected through population-based surveys such as the European Labour Force Survey and the NESCaV (Nutrition, Environment and Cardiovascular Health) study. Secondary data was obtained from open and closed data sources, e.g., from health insurance companies, governments, national health ministries, and other institutions. This was particularly evident in studies on COVID-19/SARS-CoV-2 and cancer survival.

### Synthesis of Results

A detailed summary of the results is presented in Table 1.

**TABLE 1:** A summarised overview of the selected studies and their characteristics (Netherlands, 2024).

ID	Study	Geographical scope	Temporal scope	Health outcome	Results	Data source
Antibiotic Resistance						
[34]	Deurenberg et al. 2005	BE, NL, DE (NUTS-3)	1999–2004	Methicillin-resistant *Staphylococcus aureus* (MRSA)	Group Q strains were predominantly SCCmec type II and ST225, with new findings in Germany revealing ST225-MRSA-II and ST241-MRSA-III, and some strains had unique SCCmec type combinations, including previously undescribed profiles, which were linked to antibiotic susceptibility	Clinical MRSA isolates from participating hospitals
[44]	Dequeker et al., 2024	BE, NL (NUTS-2)	2018–2019	Prevalance of faecal carriage of antimicrobial resistant bacteria	Prevalence of antibiotic-resistant bacteria (ESBL-E and CipR-E) was higher in Belgium compared to the Netherlands among children in daycare centers, with antimicrobial use and hospital admissions being significantly lower in the Netherlands. Risk factors included traveling to Asia and antimicrobial use, while cleaning practices helped reduce CipR-E carriage	Primary data collection of faecal samples from children in participating day care centres
[35]	Dik et al. 2016	NL, DE (postcode-level)	2010	Antibiotic prescription rate	Antibiotic prescription rates differed between primary care patients in northern Netherlands (29.8%) and north-west Germany (38.9%) and notably higher second-generation cephalosporin usage among German children (25%) compared to Dutch children (<0.1%)	Secondary data of pharmacist (NL: IADB) and health insurance (DE: BARMER GEK)
[36]	Glasner et al. 2022	NL, DE (hospital)	2017–2018	Multidrug-resistant organisms (MDRO)	MDRO prevalence, including MRSA, VRE, and 3GCRE, was higher in Germany than the Netherlands, with comparable CRE prevalence, likely influenced by distinct healthcare structures	Primary data collection of nasal and rectum swabs from participating hospitals and intensive care units
[37]	Jurke et al. 2019	NL, DE (NUTS-3)	2012–2016	MRSA	Dutch and German hospitals differed significantly, with Germany having considerably higher MRSA rates and screening rates compared to the Netherlands	Primary data collection of MRSA-surveillance data from participating hospitals using a protocol adapted from the national German Nosomial Infectious Surveillance System
[38]	Köck et al. 2009	NL, DE (hospitals)	2006 (DE), 2007 (NL)	MRSA	MRSA prevalence is higher in regional German hospitals on admission, classical risk factors are effective in identifying MRSA patients, and livestock-associated MRSA lineage ST398 is prominent in Dutch and emerging in German isolates, with frequent transmission between regional German EUREGIO hospitals	Primary data collection through nasal swabs from participating hospitals
[39]	Paget et al. 2015	NL (NUTS-2), DE (NUTS-3)	2011–2012	MRSA	The MRSA prevalence in community outpatient populations along the Dutch-German border was low, with similar livestock-associated MRSA patterns in GP patients from both countries but distinct spa types indicating healthcare-associated MRSA in German urologist outpatients	Primary data collection through nasal swabs and questionnaires by participating general practitioners (GPs)
[40]	van der Donk et al. 2012	BE, NL, DE, (hospitals)	2009–2011	Prevalence of a bacteria that carries antibiotic resistance genes	Resistance in Dutch, Belgian, and German isolates differed significantly, with Belgium having the highest overall resistance and the Netherlands the lowest, while Germany exhibited the highest prevalence of ESBL-producing isolates	Primary data collection of *E. coli* isolates from urine samples of patients from participating hospitals
[41]	Van Der Donk et al. 2013	BE, NL, DE	2009–2012	Prevalence of a bacteria that carries antibiotic resistance genes	Resistance prevalence among *E. coli* isolates in the Euroregion countries was similar, but it varied notably among different patient populations, with multiple clones identified via electrophoresis analysis, indicating the spread of resistant clones throughout the entire Euregion	Primary data collection of *E. coli* isolates from urine samples from patients attending urology services, general practitioners’ patients, and nursing home residents
[42]	Van Der Donk et al. 2013	NL, DE (nursing homes)	2009–2011	Prevalence of methicillin-susceptible *Staphylococcus aureus* (MSSA) and MRSA	*Staphylococcus aureus* prevalence was higher in German nursing homes (39%) compared to Dutch ones (30%). German MSSA isolates showed greater resistance, and MRSA rates were also higher in Germany. Two MRSA clones spread within German nursing homes, while the MSSA population structure differed significantly between the Netherlands and Germany, suggesting limited cross-border spread	Primary data collection through nasal swabs from patients residing in participating nursing homes
[43]	Zhou et al. 2017	NL, DE (hospitals)	2010–2012 (control group), 2012–2013	Prevalence of ESBL/pAmpC-Enditerobacteriaceae	Hospitals in the Northern Dutch-German region had similar prevalence of ESBL/pAmpC-Enterobacteriaceae, with slightly higher VRE rates in German hospitals, and few epidemiologically related ESBL-E. coli and VRE cases	Primary data collection through rectal swabs from hospitalized patients
COVID-19/Sars-CoV-2						
[45]	Chilla et al. 2022	DE, CZ (NUTS-3), PL, NL, CH, FR, DK, AT (NUTS-2), BE (NUTS-0)	2020–2021	Incidences of COVID-19 cases	Border incidence types were identified, including symmetric, asymmetric without spillovers, and asymmetric with spillovers, and not all border controls effectively prevented spillover effects	Secondary data from the European Centre for Disease Prevention and Control, and the Swiss Federal Office of Public Health (Open data Source)
[48]	Grimée et al. 2022	CH, IT (NUTS-2)	2020	COVID-19 cases	A counterfactual scenario of no Swiss-Italian border closure would have nearly doubled cumulative cases, while an earlier border closure only slightly reduced cases and delayed the epidemic by a few weeks	Secondary data from the Federal Office of Public Health for Swiss data and Presidency of the Council of Ministers - Civil Protection Department for Italian data
[46]	Mertel et al. 2023	DE, CZ	2021	COVID-19 cases	The border showed an overall inhibitory effect, with stronger inhibition from Saxony to Czechia, marked spatial variation in disease spread inhibition along the border, and the Löbau area in Saxony emerged as a hotspot for cross-border disease transmission	Secondary data from the Saxony State Government for German data and the Ministry of Health of the Czech Republic for Czech data
[47]	Serwin et al. 2022	DE (NUTS-1), PL (NUTS-3)	2021	SARS-CoV-2 transmission	Among non-Alpha lineages, 5.05% were binational clusters, 86.63% were German, and 8.32% were Polish; for B.1.1.7|Alpha variants, 13.11% were binational, 68.44% German, and 18.45% Polish, with transmission hubs in Saxony, West Pomerania, and Lower Silesia, reflecting viral dynamics in the border area, crucial for informing cross-border pandemic intervention policies	Secondary data of SARS-CoV-2 sequences by the Global Initiative on Sharing All Influenza Data database (GISAID)
Further Infectious Disease						
[49]	Kozińska et al. 2016	PL, CZ (NUTS-2), SK (NUTS-3)	2007–2011	*Mycobacterium tuberculosis*	Identifies six potential tuberculosis transmission outbreaks among patients of different nationalities but no clear epidemiological links, and the incidence of tuberculosis in Poland did not significantly affect the incidence in the Czech Republic or Slovakia	Primary data collection of microbiological data by laboratory staff, and patient documentation by clinicians from patients treated in participating healthcare centres
[50]	Stefanoff et al. 2014	CZ, PL (district-level)	1999–2008	Tick-Borne Encephalitis and Lyme Borreliosis	Significant variations in disease incidence exist between neighbouring Czech Republic and Poland, persisting even after adjusting for natural disease gradients and population density, implying potential differences in surveillance system performance due to administrative borders not hindering zoonotic disease transmission	Secondary data by the National Institute of Public Health in Prague for Czech data and the National Institute of Public Health – National Institute of Hygiene in Warsaw for Polish data
Cancer Survival						
[52]	Rudolph et al. 2021	DE, DK (NUTS-2)	2004–2013	Breast cancer survival	Significant regional differences in breast cancer survival exist, with worse outcomes in Southern Denmark and Zealand compared to Schleswig-Holstein, largely attributed to variations in stage distribution and treatment administration, although these differences are expected to diminish in the future with Denmark’s national screening program and increased adjuvant cancer therapy usage	Secondary data by the Schleswig-Holstein Cancer registry for German data, and the Nordic statistical database NORDCAN and the Danish hospital for Danish data
[53]	Rudolph et al., 2023	DE, DK (NUTS-2)	2004–2016	Colorectal cancer survival	While colorectal cancer survival improved in both the German and Danish regions from 2004 to 2016, the improvement was greater in Denmark. By 2014–2016, colon cancer survival was similar across regions, but rectal cancer survival was significantly better in Denmark	Secondary data by the Schleswig-Holstein Cancer registry for German data, and the Nordic statistical database NORDCAN and the Danish hospital for Danish data
[51]	Storm et al. 2015	DE (NUTS-2), DK (NUTS-2)	2004–2006, 2007–2009	Colorectal cancer survival	Rectal cancer incidence and mortality rates were similar for both genders, though slightly higher in Zealand. In contrast, colon cancer was more common in Zealand, with significant differences. However, there were data quality issues in Schleswig-Holstein, highlighting the need for better patient information registration	Secondary data from the Cause of Death Register, Central Population Register and National Patient Register for Danish data, and the Statistical Office and Local Health Authorities for German data
Other health outcomes						
[56]	Alkerwi et al. 2015	LU (NUTS-0), BE (NUTS-1), FR (NUTS-2)	2008–2012	Physical Activity	Luxembourg had the highest adherence to physical activity recommendations (82%), with gender differences indicating more inactive women, while Lorraine and Wallonia had lower adherence compared to Luxembourg	Primary data collection through a population-based survey carried out by the NESCaV study (Nutrition, Environment and Cardiovascular Health)
[55]	Alonso-Sardón et al., 2023	ES – PT (district-level)	2020	Prevalence of neurodegenerative diseases	Neurodegenerative diseases affected 1.85% of the population in the Spanish-Portuguese rural border region in 2020, with higher prevalence in Salamanca, Spain (2.51%), compared to Bragança (1.87%) and Guarda (1.66%) in Portugal. The prevalence was higher among females in both countries	Secondary data collection from regional health authorities. Electronic Clinical Record of Primary Care in Spain and Sistema de Informacao das Administraciones Regionais de Saude in Protugal
[54]	Nonnenmacher et al. 2021	FR (NUTS-3), BE, DE, LU, CH	2013–2018	Perceived health and Physical health factors	Cross-border workers (CBWs) are generally healthier, with health disparities varying among CBW groups based on work destinations (with commuters to Luxembourg exhibiting the best health outcomes and those toward Germany the worst), suggesting that the spill over phenomenon assumption is not supported, and these disparities are more related to labour status than demographics	Primary data collection through Enquete Emploi, a French survey segment of the European Labour Force Survey
[57]	Fries et al. 2007	BE, NL, DE (NUTS-3)	2002	Cardiopulmonary resuscitation (CPR) outcomes	CPR outcomes are similar among neighboring EMS systems, but neurological outcomes are influenced by various factors, and cross-border CPR assistance needs enhancement	Primary Data collection through protocol screening of EMS systems

#### Antibiotic Resistance

The selected studies on antibiotic resistance of our scoping review are clustered in the border region of Germany [34–43], Netherlands [34–44] and Belgium [34, 40, 41, 44]. These investigations delve into the epidemiology of various antibiotic-resistant pathogens, including *Escherichia coli* (*E.coli*) [40, 41, 43], methicillin-resistant *Staphylococcus aureus* (MRSA) [34, 36–39, 42], vancomycin-resistant *Enterococcus faecium*/*E. faecalis* (VRE) [36, 43, 44], ciprofloxacin-resistant Enterobacterales (CipR-E) [44], extended-spectrum beta-lactamase-producing Enterobacterales (ESBL-E) [40, 43, 44], third-generation cephalosporin-resistant (3GCRE) [36], carbapenemase-producing *Enterobacterales* (CPE) [44], and carbapenem-resistant *Enterobacteriales* (CRE) [36]. An additional paper [35] examined the prevalence of antibiotic prescriptions among primary care patients. It found that the proportion of German patients receiving at least one antibiotic was higher than that among Dutch patients, particularly among children. While one study finds similar MRSA prevalence and types among outpatients of general practitioners in Germany and the Netherlands [39], many studies show heterogeneities in multidrug resistance among the three countries. Germany shows a higher prevalence of resistance in MRSA [36–38, 42], VRE [36, 43] and CRE [36] compared to the Netherlands among intensive care patients [36], inpatients [37, 38, 43] and nursing home residents [42]. These differences can, among other things, be attributed to variations in healthcare structures and systems, such as higher antibiotic prescription rates [35], higher hospitalisation rates, and longer hospital stays [37]. Furthermore, variations in the examination of MRSA rates occurred within hospitals and even departments [38]. Additionally, the prevalence of multi-drug resistant *E.coli* isolates varied significantly in the German-Dutch-Belgian border region among different patient populations, with Belgium showing the highest prevalence and the Netherlands the lowest [40]. Another study in the same region reveals comparable *E.coli* resistance prevalence among the three countries but significant differences among various patient groups, with the highest prevalence observed in urology [41]. One study compares the border regions of the Netherlands and Belgium and finds a higher prevalence of ESBL-E and CipR-E in children attending daycare centres on the Belgian side [44]. Most of these studies lack additional clinical and patient information, primarily due to data availability issues, particularly concerning comorbidities.

#### COVID-19/SARS-CoV-2

Since 2020, studies examining COVID-19 with a focus on border-related aspects have emerged, primarily in the border regions of Germany and its neighbouring countries [45–47] and at the border regions of Switzerland and Italy [48]. These investigations aim to understand border incidence types and the effectiveness of border controls in mitigating spillover effects. The research findings reveal distinct patterns across different countries. A study establishes a typology by dividing neighbouring country pairs into symmetric and asymmetric pairs [45]. Symmetric pairs, such as DK-DE during the first wave, exhibit similar infection rates and trends on both sides of the border, suggesting that the border does not significantly impact infection dynamics. In contrast, asymmetric pairs without spillover effects, like BE-DE during the second wave, display significant differences in infection rates and their change over time, indicating the effectiveness of containment measures in preventing spillover. Furthermore, asymmetric pairs with spillover effects, exemplified by CZ-DE during the second wave, show varying infection rates that eventually converge over time, albeit with a time lag. This suggests the presence of spillover effects, particularly in German border regions [45]. Interestingly, an inhibitory effect of borders on COVID-19 transmission was observed in the German-Czech border region, although this effect is asymmetrical, with stronger inhibition from Germany to the Czech Republic than in the reverse direction [46]. Moreover, two specific hotspots for cross-border SARS-CoV-2 virus spread were identified. One hotspot encompassed the cross-border region including Saxony in Germany and West Pomerania as well as Lower Silesia in Poland, spanning the period from 2020 to 2021 [47]. Another hotspot was identified in the German-Czech border region [46]. Lastly, the impact of border closure on the prevalence of COVID-19 cases in the Swiss-Italian border region was quantified [48]. Counterfactual scenarios were modelled, revealing that the absence of border closure would have nearly doubled the cumulative cases of COVID-19 infections. An earlier border closure, although only slightly reducing cases, did manage to delay the epidemic by a few weeks.

#### Other Infectious Diseases

Beyond the extensive research on antibiotic resistance and COVID-19/SARS-CoV-2, additional studies have explored various infectious diseases [49, 50]. Notably, a transmission outbreak of Tuberculosis involving patients from different nationalities was detected in the Polish-Czech-Slovakian border region [50]. However, no epidemiological link was established, and incidences of tuberculosis in Poland did not significantly influence the incidence in neighbouring areas across the border. In a separate study encompassing the entire border region of the Czech Republic and Poland, the focus was on zoonotic diseases, specifically tick-borne encephalitis (TBE) and Lyme borreliosis (LB) [49]. Findings indicate persistent differences in disease incidence between these neighbouring countries. The Czech Republic exhibited a higher risk ratio for TBE and LB incidences, even after adjusting for epidemiological gradients and population density across the regions. This observation highlights the presence of substantial variations in surveillance systems between the two countries, suggesting that administrative borders alone do not fully account for the patterns of zoonotic diseases.

#### Cancer Survival

In cross-border research within the Fehmarn Belt region, which encompasses the state of Schleswig-Holstein in Germany and the region Zealand [51] in Denmark, along with the region of Southern Denmark [52, 53], two studies have illuminated regional disparities in cancer survival, with more favourable outcomes observed among German patients. This overarching theme of regional variations is particularly pronounced in cases of breast and colon cancer. Specifically, breast cancer patients in the Danish border region exhibited significantly lower overall cancer survival rates compared to their counterparts in the German border regions [52]. These differences appear to stem from variations in cancer screening and treatment protocols. Germany initiated breast cancer screening and adjuvant cancer therapy earlier than Denmark. However, the implementation of national cancer screening and therapy plans by Denmark is viewed as a potential means to narrow the cancer survival gap between the regions. Similar patterns emerged in the case of colon cancer patients [51], with Schleswig-Holstein showing a lower incidence rate, lower mortality rate, and higher survival rate compared to Zealand. However, the disparities were less pronounced among rectum cancer patients. The study was conducted again with updated data, which showed that the Danish border region caught up with and even surpassed the German border regions [53]. By the end of the observation period, survival rates for colon cancer were similar across the border, while survival rates for rectal cancer were higher in Danish border regions. Concerns were raised regarding data availability and, consequently, the comparability of cancer survival between the two countries. Notably, cases in Germany were primarily known from death certificates, and information regarding co-morbidities or socio-economic characteristics of the study population was lacking in both studies.

#### Other Health Outcomes

Studies focusing on other health outcomes were found in French [54], Spanish-Portuguese [55], Belgian-Luxembourgish-French [56] and Dutch-German-Belgian [57] border regions. Cross-border workers (CBWs) have better health outcomes and higher income compared to non-cross-border workers (NCBWs), showing a strong positive correlation between income and health [54]. In this paper, health is measured from five self-reported health variables, i.e., low perceived health, activity limitation, chronic diseases, disabilities, and lack of leisure activities. The disparities of these combined health outcomes among CBWs are linked to work destinations. Commuters to Luxembourg reported better self-reported health while commuters to Germany reported the worst. In another study, it was shown that individuals living in Luxembourg have a higher likelihood of meeting the physical activity recommendation, defined by the World Health Organisation, compared to the neighbouring regions, Wallonia in Belgium and Lorraine in France [56]. In the border regions between Spain and Portugal, variations in the prevalence of neurodegenerative diseases have been observed [55]. The prevalence is higher in the Spanish border region compared to the Portuguese counterpart. Furthermore, the prevalence in the entire cross-border region exceeds the European and global averages, presumably attributed to the rurality of the region and its significant elderly population. Lastly, outcomes of cardiopulmonary resuscitation were similar in Belgium, the Netherlands and Germany, even though medical and organisational differences among the three emergency medical services exist [57]. Furthermore, cross-border emergency assistance for cardiopulmonary resuscitation was hardly identified, which could be related to legal and communication obstacles.

## Discussion

This scoping review searched for trends and differences in health outcomes in cross-border regions within the EU. It uncovered 785 distinct studies, of which only 24 met our eligibility criteria. Most of the studies were excluded due to wrong geographical settings, health outcomes, research objectives or study design. Based on the health outcomes analysed by the included studies, we identified five thematic and geographic clusters: Antibiotic resistance, COVID-19/SARS-CoV-2, other infectious diseases, cancer survival, and other health outcomes. While the setting of EU border regions is mainly used to investigate various infectious diseases transmitted from one country to another, macro health levels and trends, such as mortality and morbidity outcomes, are sparsely represented in the literature corpus.

We have discussed the health differences of cross-border regions that resulted from the selected studies. However, we know very little about how these differences in health outcomes developed over time. Moreover, no references are being made to how health trends relate to EU integration processes. This is due to two reasons; First, we did not find any study that started its observation period at the foundation of the EU in 1992 when the Maastricht Treaty was signed. The data collected only goes back to 1999. Second, most studies are cross-sectional rather than longitudinal, which means that the health parameters are rarely observed over multiple years. Studies on COVID-19 do examine the incidence rates and changes over time, but due to the recent emergence of the virus, the study period is rather short. As an exception, three studies are looking at the trends of health outcomes for a longer period [50, 52, 53].

In addition to the temporal limitations of the studies, we have identified gaps in the spatial coverage. Almost all studies are focused on a particular cross-border region of two or three countries. Only one study has compared the outcomes of German border regions with those of all its eight neighbouring countries. Moreover, the study regions of the articles are mainly clustered among Central and Western European countries. Specifically, German, Dutch, Belgian, and French border regions are well represented in this scoping review. However, we noticed a lack of studies on border regions from Eastern, Southern and Northern European countries, as well as a comprehensive overview of multiple, or even all member states.

Studies on infectious diseases mostly use the cross-border setting to detect their spread across borders, due to the high cross-border mobility of patients, workers, and tourists. Especially, studies on antibiotic resistance do address that the border region of Germany and the Netherlands may serve as a “living lab,” stressing the importance of the different healthcare systems, practices, protocols, and surveillance in the healthcare units on both sides of the border. Interestingly, although not its main focus, one study showed that living closer to the border seems to increase antibiotic consumption, which the authors explain by the fact that cross-border regions are further away from the medical centre of the respective countries and exhibit lower socioeconomic status, both of which are associated with antibiotic consumption [35]. This outcome is a minor side result, mentioned only briefly in the text and not listed as a main result. Yet, we do not know how opening the border affected the prevalence of different antibiotic resistance of the patients in those cross-border regions. Furthermore, in the studies on cancer survival [51–53] and adherence to physical activity recommendations [56], regional comparisons rarely yield anything specific about cross-border regions and their populations as such. Instead, it seems that they are used to compare countries, making border regions merely placeholders for the entire country. The only study that appears to directly address cross-border populations as distinct is by Nonnenmacher and colleagues [54] who compared several health outcomes of cross-border workers and non-cross-border workers.

A potential reason for the research gaps is the lack of cross-border data that is both available at a regional level and comparable across all member states. Countries may have different definitions and registration systems in their healthcare systems, making it challenging to gather comparable data. This was particularly evident in the studies on cancer survival [51–53] in Denmark and Germany, which addressed the different registrations of cancer in the respective countries. As a result, caution is required when interpreting the survival rates.

This review emphasises the need to strengthen infrastructure for data sharing and collaboration between border regions. There are some positive developments in this area, such as the establishment of a regional health atlas by Meuse-Rhine-Euroregion, which covers indicators on mortality and healthcare resources [58]. Additionally, the Cross-Border Institute of Healthcare Systems and Prevention is creating a health atlas and data inventory for the Ems-Dollard Region to facilitate cross-border research. While these initiatives demonstrate how localized efforts can inform broader policy frameworks, it is essential to scale such projects across all member states to ensure equitable and effective health interventions. Eurostat published an atlas on mortality statistics in the EU in 2002 [59], which was updated with new data in 2009 [60]. However, there have been no further updates since then. Although open data sources like Eurostat and the Atlas of Population Health in European Union Regions [61], provide useful overviews of regional health in the EU, they either date back 10 years or are limited to NUTS-2 regions, making it difficult to identify border regions. Robust cross-border policies are needed, similar to those envisioned in the European Health Union initiative, to mitigate these challenges. Such policies would foster standardised data collection and uniform reporting mechanisms, as well as facilitate the use of this data at a subnational level.

### Limitations

This scoping review also comes with limitations. Since we restricted our search to English publications exclusively, there is a possibility that we might have left out pertinent studies that were published in a different language and could have been relevant to our research. This may be especially relevant for national reports published in the country’s official language. For example, publications regarding COVID-19 cases, such as the report on the impact of the COVID-19 crisis in German border regions by the Federal Office for Building and Regional Planning [62], or studies on cross-border infection threats in border areas, may have been excluded from this study. Furthermore, we limited our scope to published studies and reports. While this approach is common practice in literature reviews, it may result in a publication bias, as unpublished or grey literature was not considered. Ultimately, our review focused exclusively on health outcomes of the population. Consequently, we did not consider studies addressing socio-economic factors and determinants of health, which could additionally explain our lack of findings related to health trends and European integration. Including socio-economic factors in our search, however, was beyond the scope of our research. Our primary objective was to compile literature on the differences in health outcomes among European cross-border regions at a macro level. Moreover, focusing on population health determinants and contextual factors could be a promising addition to this work in the future, as they could provide deeper insights into the underlying mechanisms of cross-border health disparities and trends.

### Conclusion

In this scoping review, we identified temporal, geographical, and thematic gaps in the literature. The studies found were predominantly epidemiological, cross-sectional studies covering a specific border region. Research on infectious diseases, such as antibiotic resistance and, more recently, on COVID-19, was particularly dominant. Meanwhile, studies on macro-level health trends in cross-border regions were lacking in the scanned literature. This is a significant shortcoming in the existing literature since cross-border regions could act as “living labs” for evaluating the effectiveness of European integration. Additionally, health profiles of cross-border regions can serve as benchmarks for measuring successful regional convergence of living standards. Therefore, more health-relevant data and research are needed on health trends and their underlying determinants to identify best practices that can aid policymaking, not just in the health domain but also for those concerned with European integration.
