# Supplementary material for: Health Outcomes in EU Cross-Border Regions: A Scoping Review
Source: Public Health Rev. 2025 Feb 24;46:1608170. doi: 10.3389/phrs.2025.1608170 (PMC11891012; doi:10.3389/phrs.2025.1608170)
Supplement: Supplementary file 1 [file DataSheet2.DOCX]

# Supplementary Material: Syntax and Key Terms

**Key terms**

|  | **Cross-border** | **European Union** | **Health Outcome** |
| --- | --- | --- | --- |
| Index Terms | - | European Union | Life Expectancy, mortality, morbidity |
| Keywords | Cross-border, border region, border area | European Union, member state, Europe, European Integration, Euroregio,  + all country names and languages (e.g. The Netherland, Dutch) | Health outcome, death rate, survival rate, life table  Diseases, prevalence, incidence, basic reproduction number |

Pubmed Syntax:

(“cross-border*”[tiab] OR “cross border*”[tiab] OR “border* region*”[tiab] OR “border* area*”[tiab]) AND (“European Union”[MesH] OR “european union”[tiab] OR “eu”[tiab] OR “member state*”[tiab] OR “european”[tiab] OR “europe”[tiab] OR “european integration”[tiab] OR “european community”[tiab] OR “euroregio*”[tiab] OR austria* [tiab] OR belgium [tiab] OR belgian[tiab] OR bulgaria*[tiab] OR croatia*[tiab] OR cyprus[tiab] OR cypriot[tiab] OR “czech republic”[tiab] OR czech*[tiab] OR denmark[tiab] OR danish[tiab] OR estonia*[tiab] OR finland[tiab] OR finnish[tiab] OR france[tiab] OR french[tiab] OR german*[tiab] OR greece[tiab] OR greek[tiab] OR hungar*[tiab] OR ireland[tiab] OR irish[tiab] OR italy[tiab] OR italian[tiab] OR latvia*[tiab] OR lithuania*[tiab] OR luxembourg*[tiab] OR malta[tiab] OR maltese[tiab] OR netherlands[tiab] OR dutch[tiab] OR norway[tiab] OR norwegian[tiab] OR poland[tiab] OR polish[tiab] OR portugal[tiab] OR portuguese[tiab] OR romania*[tiab] OR slovak*[tiab] OR sloven*[tiab] OR spain[tiab] OR spanish[tiab] OR sweden[tiab] OR swedish[tiab] OR switzerland[tiab] OR swiss[tiab]) AND (“life expectancy”[Mesh] OR “mortality”[MesH] OR “morbidity”[MesH] OR “life expectanc*”[tiab] OR “mortalit*”[tiab] OR “morbidit*”[tiab] OR “health outcome*”[tiab] OR “healthy life expectanc*”[tiab] OR “death” [tiab] OR “life table*”[tiab] OR “survival” [tiab] OR “disease*”[tiab] OR “prevalence”[tiab] OR “incidence”[tiab] OR “basic reproduction number”[tiab])

Syntax Scopus

(TITLE-ABS-KEY("cross-border*" OR "cross border*" OR "border* Pre/2 region*" OR "border* Pre/2 area*") AND TITLE-ABS-KEY("european union" OR "eu" OR "member state" OR "european" OR "europe" OR "european integration" OR “euroregio*” OR austria* OR belgium OR belgian OR bulgaria* OR croatia* OR cyprus OR cypriot OR "czech republic" OR czech* OR denmark OR danish OR estonia* OR finland OR finnish OR france OR french OR german* OR greece OR greek OR hungar* OR ireland OR irish OR italy OR italian OR latvia* OR lithuania* OR luxembourg* OR malta OR maltese OR netherlands OR dutch OR norway OR norwegian OR poland OR polish OR portugal OR portuguese OR romania* OR slovakia OR slovak OR slovenia OR slovene OR spain OR spanish OR sweden OR swedish OR switzerland OR swiss) AND TITLE-ABS-KEY("health outcome*" OR "life expectanc*" OR "healthy life expectanc*" OR "mortalit*" OR “morbidit*” OR "death rate" OR "life table*" OR “survival” OR “disease*” OR “prevalence” OR “incidence” OR “basic reproduction number”))

Syntax Web of Science

TS=(("cross-border*" OR "cross border*" OR "border* NEAR/2 region*" OR "border* NEAR/2 area*") AND ("european union" OR "eu" OR "member state" OR "european" OR "europe" OR "european integration" OR “euroregio*” OR austria* OR belgium OR belgian OR bulgaria* OR croatia* OR cyprus OR cypriot OR "czech republic" OR czech OR denmark OR danish OR estonia* OR finland OR finnish OR france OR french OR german* OR greece OR greek OR hungar* OR ireland OR irish OR italy OR italian OR latvia* OR lithuania* OR luxembourg* OR malta OR maltese OR netherlands OR dutch OR norway OR norwegian OR poland OR polish OR portugal OR portuguese OR romania* OR slovakia OR slovak OR slovenia OR slovene OR spain OR spanish OR sweden OR swedish OR switzerland OR swiss) AND ("health outcome*" OR "life expectanc*" OR "healthy life expectanc*" OR "mortalit*" OR “morbidit*” OR "death rate" OR "life table*” OR “survival” OR “disease*” OR “prevalence” OR “incidence” OR “basic reproduction number”))

Socindex (EBSCOhost)

(TI (“cross-border*” OR “cross border*” OR “border* region*” OR “border* area*”) OR AB (“cross-border*” OR “cross border*” OR “border* region*” OR “border* area*”)) AND (MH “European Union”+ OR TI (“european union” OR “eu” OR “member state*” OR “european” OR “europe” OR “european integration” OR “european community” OR “Euroregio*” OR Austria* OR Belgium OR Belgian OR Bulgaria* OR Croatia* OR Cyprus OR Cypriot OR “Czech Republic” OR Czech OR Denmark OR Danish OR Estonia* OR Finland OR Finnish OR France OR French OR German* OR Greece OR Greek OR Hungar* OR Ireland OR Irish OR Italy OR Italian OR Latvia* OR Lithuania* OR Luxembourg* OR Malta OR Maltese OR Netherlands OR Dutch OR Norway OR Norwegian OR Poland OR Polish OR Portugal OR Portuguese OR Romania* OR Slovakia OR Slovak OR Slovenia OR Slovene OR Spain OR Spanish OR Sweden OR Swedish OR Switzerland OR Swiss) OR AB (“european union” OR “eu” OR “member state*” OR “european” OR “europe” OR “european integration” OR “european community” OR “Euroregio*” OR Austria* OR Belgium OR Belgian OR Bulgaria* OR Croatia* OR Cyprus OR Cypriot OR “Czech Republic” OR Czech OR Denmark OR Danish OR Estonia* OR Finland OR Finnish OR France OR French OR German* OR Greece OR Greek OR Hungar* OR Ireland OR Irish OR Italy OR Italian OR Latvia* OR Lithuania* OR Luxembourg* OR Malta OR Maltese OR Netherlands OR Dutch OR Norway OR Norwegian OR Poland OR Polish OR Portugal OR Portuguese OR Romania* OR Slovakia OR Slovak OR Slovenia OR Slovene OR Spain OR Spanish OR Sweden OR Swedish OR Switzerland OR Swiss)) AND (MH “life expectancy”+ OR MH “mortality”+ OR MH “morbidity”+ OR TI (“health outcome*” OR “life expectanc*” OR “healthy life expectanc*” OR “mortalit*” OR “morbidit*” OR “death rate” OR “life table*” OR “survival” OR “disease*” OR “prevalence” OR “incidence” OR “basic reproduction rate”) OR AB (“health outcome*” OR “life expectanc*” OR “healthy life expectanc*” OR “mortalit*” OR “morbidit*” OR “death rate” OR “life table*” OR “survival” OR “disease*” OR “prevalence” OR “incidence” OR “basic reproduction rate”))
